# Incidental Findings in Clinical Practice: A Case of Hepatic Encephalopathy and Subsequent Lung Cancer Detection

**DOI:** 10.7759/cureus.88245

**Published:** 2025-07-18

**Authors:** Evan J Ernest, Peter Shih

**Affiliations:** 1 Internal Medicine, Edward Via College of Osteopathic Medicine, Auburn, USA; 2 Pulmonary and Critical Care Medicine, HCA Florida Fort Walton-Destin Hospital, Fort Walton Beach, USA

**Keywords:** alcoholic liver cirrhosis, altered mental state, endobronchial ultrasound (ebus), hepatic encephalopathy, incidental radiological finding, non-small cell lung carcinoma (nsclc), robotic bronchoscopy

## Abstract

Altered mental status (AMS) poses a challenge for healthcare providers due to its extensive differential diagnosis. A 67-year-old female patient with a complex medical history was admitted to the hospital due to AMS stemming from hepatic encephalopathy. During her evaluation, a routine chest X-ray revealed a right upper lobe pulmonary nodule, which was subsequently diagnosed as non-small cell lung cancer following further imaging and biopsy. This case emphasizes the importance of comprehensive assessments of incidental findings. The unanticipated identification of lung cancer in this instance underscores the critical role of early detection and a holistic diagnostic strategy, especially in populations at heightened risk, to inform effective management and enhance patient outcomes.

## Introduction

Altered mental status (AMS) represents a significant clinical syndrome characterized by changes in cognitive and psychological function, frequently observed in the elderly. This condition poses a challenge for healthcare providers due to its extensive differential diagnosis. AMS often arises from underlying medical issues that can be life-threatening, leading to various unfavorable outcomes [1]. The pathophysiologic changes seen with aging, the higher occurrence of multiple comorbidities, and the greater need for medications are all contributing factors to AMS seen in elderly patients [2]. Among the various causes of AMS, hepatic encephalopathy is frequently encountered in patients with liver disease, particularly cirrhosis [3]. Hepatic encephalopathy causes decreased mental function and reversible neuropsychiatric abnormalities [4], such as hyperreflexia, rigidity, bradykinesia, myoclonus, and asterixis, resulting from the accumulation of neurotoxic substances, such as ammonia, due to impaired liver function [5]. While initial evaluations typically reveal the primary cause of AMS, incidental findings, especially those discovered through imaging, can unveil additional, potentially serious conditions.

Incidental pulmonary nodules are being detected more frequently as a result of routine medical examinations, with their occurrence now found to be significantly higher than previously acknowledged [6]. The incidental discovery of lung nodules in patients without pulmonary symptoms is regarded as a frequent occurrence. Although the majority of incidental pulmonary nodules are benign, a portion of them can indicate early-stage lung cancer. Lung cancer, particularly non-small cell lung cancer (NSCLC), is a leading cause of cancer-related mortality worldwide [7], making further evaluation crucial. The management of incidental lung findings remains a contentious issue, with varying guidelines advising on the necessity for monitoring versus further evaluation [8]. It is essential to establish the most appropriate management strategies to prevent unnecessary invasive procedures for benign nodules while also avoiding delays in diagnosing malignant ones. The early identification of malignancy, even when discovered incidentally, can profoundly influence patient management and prognosis.

This case report details a patient presenting with AMS due to hepatic encephalopathy, and an incidental finding of a solitary pulmonary nodule that was later confirmed as NSCLC. This scenario underscores the importance of meticulous evaluation of all findings, including those discovered incidentally, to ensure comprehensive patient care.

## Case presentation

A 67-year-old woman was admitted to the hospital via emergency medical services (EMS) due to AMS. Family members reported a gradual decline in her mental state over the preceding days, culminating in her being found at home in a somnolent and altered state, exhibiting incontinence of stool and urine. The patient was a current smoker, with a history of over 50 pack-years, alongside a medical history marked by alcoholic cirrhosis, hepatitis C, and severe anemia.

On examination, the patient was alert but disoriented to time and place, lethargic, and unable to engage in a coherent conversation. Neurologically, she was confused and slow to respond, though no focal deficits were noted. Cardiovascular examination revealed a blood pressure of 120/57 mmHg, a pulse of 70 bpm, and a respiratory rate of 20 rpm, with no murmurs detected. Peripheral edema was observed in all four extremities. Respiratory examination showed clear lung fields and no signs of respiratory distress, though clubbing was noted. Abdominal examination revealed distension, caput medusae, splenomegaly, and flank dullness on percussion.

Initial laboratory investigations revealed respiratory alkalosis, indicative of cirrhosis [9], along with microcytic anemia, thrombocytopenia, hyponatremia, hyperglycemia, elevated ammonia levels and liver enzymes, and urinalysis showed epithelial cells and leukocyte esterase. The elevated ammonia, combined with her clinical symptoms, confirmed the diagnosis of hepatic encephalopathy. The patient was admitted for close monitoring and management, receiving lactulose to reduce ammonia absorption and rifaximin once her mental status improved. The patient was additionally started on empirical treatment for a suspected urinary tract infection with ceftriaxone, diuretics to manage peripheral edema, and her vital signs and laboratory results were carefully monitored.

**Figure 1:**
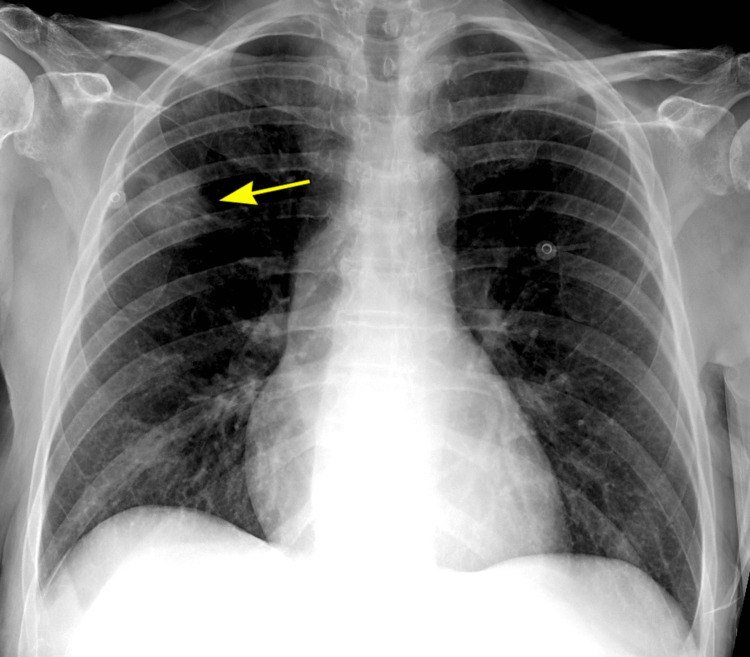
Chest X-ray showing the patient’s right upper lobe mass concerning for malignancy (yellow arrow)

**Figure 2:**
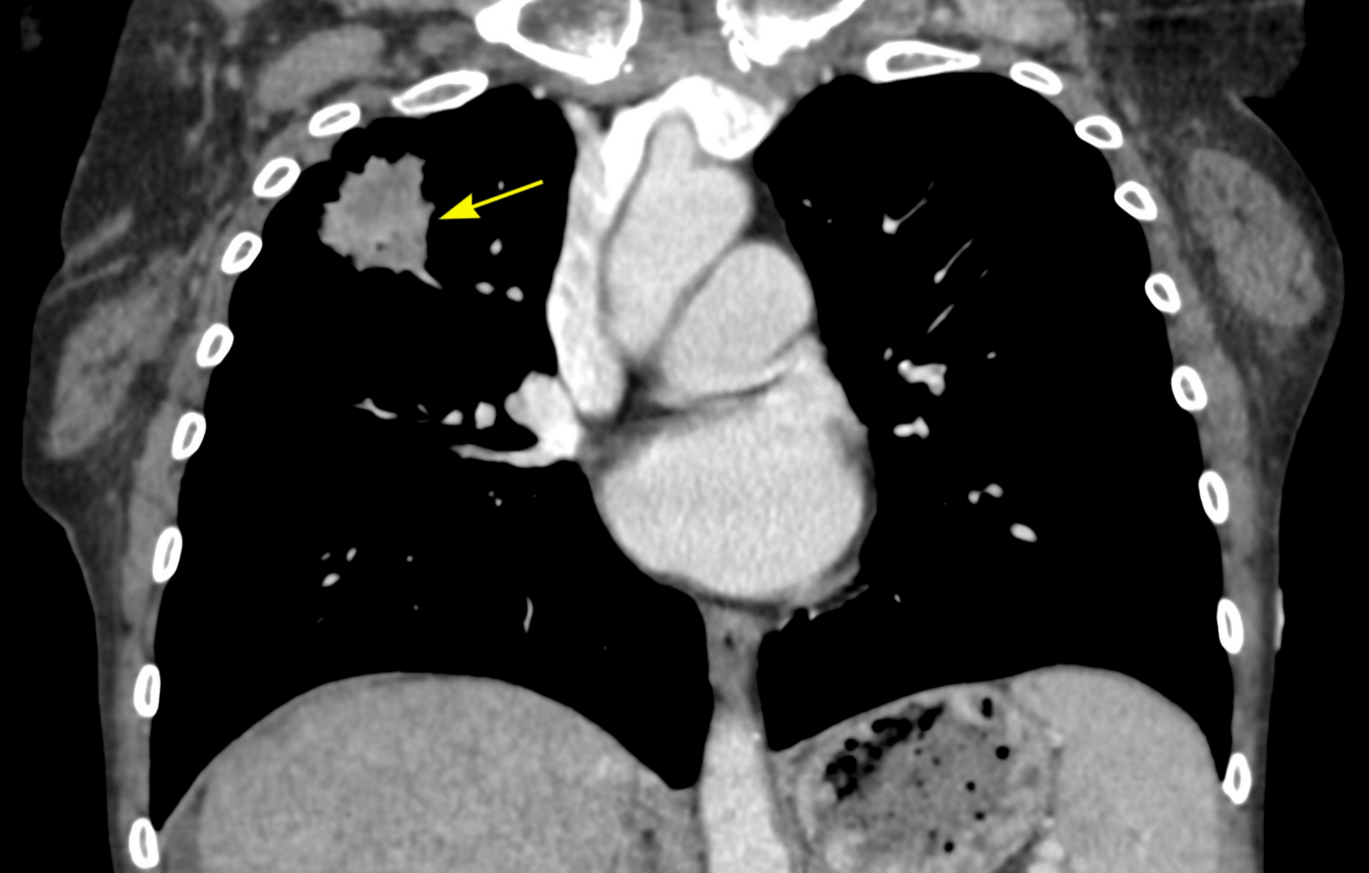
Chest CT (coronal) with contrast showing the 4-cm right upper lobe mass, compatible with malignancy (yellow arrow)

Due to her history of cirrhosis, a chest X-ray was performed to evaluate for potential pulmonary complications common in liver disease [10]. The chest X-ray revealed a right upper lobe pulmonary mass that was noted to have irregular margins (Figure 1). This finding raised concern for a malignant lesion, although it was incidental to the primary reason for her admission. Figure 2 shows a subsequent contrast-enhanced chest CT scan performed to further evaluate the pulmonary nodule, which confirmed the presence of a 4-cm spiculated mass in the right upper lobe, consistent with a primary lung malignancy. Following consultation with the pulmonary team, the patient and her family were informed of the findings and the indications, contraindications, and complications of biopsying the lung mass.

Robotic-assisted navigational bronchoscopy and endobronchial ultrasound were performed to obtain a definitive diagnosis. Histopathological analysis showed atypical epithelial cells with enlarged hyperchromatic nuclei and irregular nuclear contours suspicious for NSCLC​. The cells were positive for CK7 and TTF-1 and negative for p40, likely representing fragments of a poorly differentiated adenocarcinoma​.

## Discussion

Hepatic encephalopathy is a recognized complication of cirrhosis, driven by the accumulation of neurotoxins such as ammonia, which are normally detoxified by the liver. In cirrhosis, this process is impaired, leading to the neuropsychiatric symptoms associated with encephalopathy, including confusion, altered consciousness, and cognitive dysfunction [11]. The diagnosis of AMS in elderly patients can be particularly challenging due to the broad differential diagnosis and the potential overlap with other comorbidities, such as cirrhosis in this case [2]. Elevated ammonia levels and the patient's clinical presentation confirmed hepatic encephalopathy, which was appropriately managed with lactulose and rifaximin. However, it was the incidental pulmonary nodule detected on chest imaging that shifted the focus of her care and ultimately led to the diagnosis of NSCLC.

An incidental finding, or incidentaloma, refers to abnormalities discovered unintentionally through imaging tests performed for reasons unrelated to the condition identified [8]. Incidental pulmonary nodules are increasingly being detected due to the widespread use of imaging modalities like chest X-ray and CT [12]. These nodules, while often benign, can occasionally represent early-stage malignancies, particularly in high-risk populations, such as smokers and patients with significant comorbidities. In this case, the patient’s long history of smoking (50 pack-years) placed her at higher risk of lung cancer, and the incidental finding of a right upper lobe mass prompted further diagnostic evaluation.

The management of incidental lung nodules remains a topic of debate, with differing guidelines on whether to monitor or biopsy these findings. However, when a suspicious lesion is detected, as in this case, further diagnostic evaluation is critical. The recently updated Fleischner Society guideline for the management of incidental pulmonary nodules recommends assessing patient risk factors, such as smoking history, exposures, and family history, as well as nodule risk factors, such as size, density, multiplicity, morphology, and growth [13]. Lung biopsy via bronchoscopy is indicated for a suspicious mass seen on imaging, particularly when located in the central airways, to assess malignancy, stage cancer, or diagnose other conditions such as infections or interstitial lung disease [14]. Additional indications include unexplained hemoptysis, persistent cough, or shortness of breath associated with the mass.

In this case, the incidental discovery of a pulmonary mass in a patient presenting with altered mental status due to hepatic encephalopathy highlights the critical need for comprehensive evaluation of all imaging findings. Differentiating non-small cell lung cancer subtypes, adenocarcinoma, squamous cell carcinoma, and large cell carcinoma, is crucial for guiding treatment decisions and prognostication. Adenocarcinoma, the most common subtype, is often peripheral, commonly found in non-smokers, and expresses markers like TTF-1, Napsin A, and CK7, with potential mutations in EGFR and ALK that can be targeted with specific therapies [15]. Squamous cell carcinoma, typically central, is strongly associated with smoking [15] and expresses p40 and p63 while showing low rates of EGFR mutations. Large cell carcinoma is an undifferentiated subtype, exhibiting pleomorphic cells and a lack of glandular or squamous differentiation, often displaying aggressive behavior and poor responsiveness to targeted therapies [15]. Immunohistochemistry and molecular testing, such as for EGFR mutations and ALK rearrangements, along with imaging, are essential for distinguishing these subtypes [15].

Early detection of malignancy, even as an incidental finding, can significantly influence management strategies, emphasizing the importance of thorough evaluation, especially in complex cases like those with coexisting conditions such as cirrhosis. Accurate identification of the NSCLC subtype guides therapeutic choices, including targeted therapies and immune checkpoint inhibitors, ultimately improving patient outcomes.

Our patient had a history of alcoholic cirrhosis and a smoking history of over 50 pack-years, and although her primary diagnosis was hepatic encephalopathy, the incidental discovery of a lung mass underscores the importance of a thorough and comprehensive evaluation in patients with multiple comorbidities, particularly when imaging is performed for reasons unrelated to pulmonary symptoms.

## Conclusions

This case highlights the importance of a meticulous evaluation of all findings, including incidental ones, in patients with complex presentations. The early identification of lung cancer, even when not initially suspected, can have a significant impact on prognosis and patient outcomes. Furthermore, this case underscores the need for clinicians to maintain a high index of suspicion for malignancy in patients with risk factors such as smoking history, especially when imaging studies reveal abnormal findings that could represent early-stage cancer.
